# Stigma and its impact on the families of former soldiers of the German Armed Forces: an exploratory study

**DOI:** 10.1186/s40779-018-0188-z

**Published:** 2018-11-29

**Authors:** Katrin Schuy, Simone Dors, Loni Brants, Marie Horzetzky, Gerd Willmund, Andreas Ströhle, Peter Zimmermann, Heinrich Rau, Stefan Siegel

**Affiliations:** 1 Department of Psychiatry and Psychotherapy, Campus Charité Mitte, Charité-Universitätsmedizin Berlin, 10117 Berlin, Germany; 2 Psychotraumazentrum, Military Hospital, 10115 Berlin, Germany

**Keywords:** Stigma by association, Vicarious stigma, Families, Mental illness, Veteran, German Armed Forces

## Abstract

**Background:**

Military families who have a family member with a mental illness see themselves confronted with many demands. Stigmatization is one of these challenges. Stigmatization affects not only the individual who suffers from a mental illness but also other family members via stigma by association and vicarious stigma. Stigma by association occurs when mental illness stigma spills over to individuals associated with an individual with a mental illness. Vicarious stigma describes the suffering of family members when they note the impact of stigma on their relative with mental illness. As a societal phenomenon, stigma plays out in social interactions and might therefore influence the social networks of families. It is also associated with healthcare utilization.

**Method:**

Narrative interviews were conducted with 15 family members (partners, spouses, parents and children) of former soldiers of the German Armed Forces with a service-induced mental illness. The transcribed interview data were analyzed using a thematic analysis approach, in which codes were formed and emerging themes were systemized. Relationships between stigma, the families’ reactions to it, its effects on their social relationships and its interference with their healthcare utilization were analyzed.

**Results:**

This study provides a detailed description of how relatives of former German soldiers with mental health problems experience stigma by association and vicarious stigma. Their perceptions are shown in a model that describes stigma-related attitudes, reactions to them and their effects on the social relationships of former soldiers’ families. These families felt stigmatized because of the former soldiers’ mental illness (mental illness stigma) and the military context in which it occurred (former soldier stigma). They reacted with nondisclosure, anger, acceptance and self-blame. Stigma was associated with smaller and weaker social networks that were characterized by social exclusion, self-segregation and conflicts with extended family, friends and colleagues. Stigma also affected the families’ healthcare utilization.

**Conclusions:**

Urgently needed anti-stigma campaigns, particularly in the civilian context, should address the stigmatization of both mental illness and the military participation of the families affected. They should consider the needs of both former soldiers with a mental illness and their families.

## Background

Family members with a partner, child or sibling who suffers from mental illness are confronted with many challenges [1–8]. They are more stressed, have a higher probability of mental and physical health problems [9, 10] and have lower self-esteem [11, 12]. Due to caregiving demands, they may have financial problems, engage in fewer social activities and have less contact with their extended family and friends [3]. They must often adapt their daily lives to the demands of their relative’s mental illness, and they may have to sacrifice their own professional goals and social relationships [1, 6, 7, 13, 14]. In addition to the severe negative effects that mental illness imposes on the family, many family members must live with stigmatization and discrimination because of their relative’s mental illness.

Stigma is an interactive societal process in which individuals with a certain characteristic, e.g., a mental illness, are labeled and associated with stereotypes that are attached to this label (e.g.individuals with mental illnesses are weak) [15]. This process can lead to discrimination against the stigmatized group and a loss of status [15]. Stigmatization affects not only the individual who suffers from mental illness but also the individuals who are associated with this stigmatized individual [16–19]. They might be affected by stigma via two different pathways. The first pathway is a process whereby implicit and explicit stereotypical attitudes that were originally directed at the individual with a mental illness spill over to the individual’s family and friends [16]. This phenomenon is referred to as stigma by association (SBA) and is defined as "... the process through which the companions of stigmatized persons are discredited" [16], pg. 224. As family members often have socially and/or emotionally close relationships with the individual who suffers from mental illness, they are a common target of SBA [16]. The second pathway of stigma is when relatives perceive how their family member with mental illness is stigmatized. This process is referred to as vicarious stigma and occurs because "... family members suffer when they note the impact of prejudice and discrimination on their relative with mental illness" [18], pg. 542. Thus, in daily life, many family members of relatives with mental illness face a two-pronged problem [8, 20, 21]: On the one hand, they must manage their relative’s mental illness and its associated needs and strains, e.g., caregiving, having more responsibility, facing financial difficulties, and coping with symptoms, which are often described as family burden [3, 8]. On the other hand, they also face mental illness stigma and its consequences [8, 19–21]. Therefore, stigma can be considered an additional subjective burden [6, 22] that contributes to the overall demands that mental illness inflicts on the family [3]. It also appears to predict the size of the burden that mental illness imposes on families [23].

Given that the experience of stigma is perceived as unpleasant, individuals typically attempt to make it less distressing [8, 24]. Attempts to handle stressful situations are referred to as coping [25], which may be divided into problem-focused coping (i.e., changing the situation or relationship, such as asking for help or seeking support) and emotion-focused coping (i.e., managing the negative emotions associated with stigma, e.g., acceptance, self-blame or venting) [8, 10, 24, 26]. The type of reaction that a relative uses to deal with stigma can mediate the relationship between stigma and potential negative outcomes [10]. It is therefore important to understand how relatives react to the experience of stigma.

SBA and vicarious stigma are associated with negative outcomes for affected families [2–4, 14, 17–19, 27]. Family members experience more psychological stress [10, 18, 19, 28] and a lower quality of life [14, 17, 27, 29]. Other individuals often avoid and exclude them [18]. In some cases, families avoid social contact themselves [30] to decrease the risk of stigmatization [31]. This is particularly tragic, as social support from other individuals can buffer the effects of stress-related issues and function as an additional resource for these families [32]. This demonstrates the importance of understanding how these families’ social networks are affected by stigma.

Furthermore, SBA and vicarious stigma can interfere with healthcare utilization [17, 33], as stigma concerns have been found to be the fourth-highest barrier to treatment seeking [34]. Family members fear being blamed for their relative’s mental illness [33] and anticipate or experience stigmatization from healthcare personnel [17, 35].

Although the previously discussed findings are from the general population, they also apply to military families, and the situation for these families might be even more severe [36]. Even before mental illness strikes, the family must cope with difficult situations: A soldier’s deployment puts unique strains on the family and is associated with psychosocial consequences for the partners at home [37]. These families experience more stress than similar families in the general population [38, 39]. Compared to nondeployed personnel, spouses with partners who are deployed experience nearly twice the stress [40]. After the soldier returns, the stress is not necessarily over, as reintegration after military service contributes to these challenges [41–43]. If the soldier returns home with a service-induced mental illness, the stress factors for these families substantially increase: reintegration can become an even bigger challenge [41], relationship satisfaction may decline, and parenting issues can arise [44]. The families’ social networks play a major role, as they can help them to cope with these burdens [45–49]. The stress factors may be associated with mental health problems in relatives [50]. In a study by Eaton et al. [50], the rate of mental health problems in military spouses at home was comparable to those of soldiers returning from combat (15.6–17.1%). However, these spouses perceived less mental health stigma than soldiers with mental health problems. Therefore, many of the spouses were willing to seek help for their mental health concerns, although some of them were confronted with organizational barriers, e.g., having no child care, not getting time off of work or having difficulty with appointment scheduling [50]. It is also important to note that family members often play an important role in the former soldier’s healthcare [41]. Marek et al. [41] found that partners of service members with a mental illness are more aware of post-traumatic stress disorder (PTSD)-related symptoms than service members, and they are also more willing to report them. They suggest that stigma might play a role in this development, as it might prevent service members from seeking help. Moreover, military culture and former soldiers’ internalization of its attributes, e.g., an emphasis on being strong and self-reliant, should be considered, as these attributes are associated with less healthcare utilization [51–54]. Overall, families of former soldiers who suffer from service-induced mental illness face many challenges, and stigma might be one challenge. To date, limited research has examined the stigma experience of family members of former soldiers and the consequences it has in different spheres of their lives [55–57].

Very few studies have addressed the burdens that service-induced mental illness poses on military families of the German Armed Forces (GAF) [58, 59]. The role of stigma is also a neglected field of research: it has been investigated in active GAF members [60] and former GAF soldiers [61]; however, the role it plays in the lives of family members of former GAF soldiers has not been researched. The GAF-centered studies on stigma and healthcare usage suggest that stigma interferes with healthcare use: In *active* soldiers, stereotypical attitudes are associated with less willingness to disclose their illness and seek help [60]. In *former* GAF soldiers, who often seek care in civilian healthcare settings, an additional stigma might play a role: research into their stigma concerns has indicated that former soldiers with mental health problems feel stigmatized because of their mental illness, as well as their military past, which is the reason for their illness [61]. Whether and how stigma also affects the social networks of GAF families has not previously been researched. The scarcity of data on stigma and its consequences, as perceived by relatives of former GAF soldiers, shows that further research is urgently needed to understand the concerns and problems that these families face. Qualitative research methods enable an understanding of how participants perceive societal phenomena and the identification of aspects that are not covered in standardized stigma questionnaires [62].

The aim of this study is to shed light on the role of stigma, particularly SBA and vicarious stigma, in German military families with a former soldier who suffers from a service-induced mental health problem. We aim to develop a model that illustrates how stigma, reactions to stigma, and the effects of stigma on social relations in German military families are interrelated. Furthermore, we aim to determine the ways in which stigma influences the healthcare utilization of these families. Research shows that familial relationships, cohabitation and gender [33] shape how stigma is perceived, how individuals react to it [18] and how consequences of it are experienced [29, 63]. To account for these differences, we analyzed the perspectives of male and female spouses/partners, parents and adult children of former soldiers with mental health problems. As the first exploratory analysis in this field, our study can provide insights into how to focus future research and how to plan and implement help for these families.

## Methods

### Recruitment and sample selection

To recruit participants, the researchers designed a website that was linked to the social media channels of the German Veterans’ Organizations and Associations, particularly those addressing friends and family members of (formerly) deployed soldiers. Potential participants could register to participate in the study on this website. Candidates were considered if they met the inclusion criteria of being a parent, child, spouse or partner of a former GAF soldier who served a minimum of 29 days abroad and suffered from a service-induced mental disorder, primarily PTSD. The process of selecting participants, with whom face-to-face narrative interviews [64, 65] were conducted, was driven by two theory-based criteria. First, a potentially large and diverse population should be reached [66]. Therefore, different types of family members (partners, spouses, parents, and children) from different parts of Germany were selected. The second criterion was theoretical saturation [67, 68], which indicates that the interview process would be terminated when further interviews no longer contributed new insights. In addition to these theory-based criteria, opportunistic criteria (e.g., accessibility of the interviewees and travel costs) were applied [69].

Twenty-seven relatives were interested in the study; however, three relatives did not meet the inclusion criteria. The researchers carried out preliminary telephone interviews with the remaining individuals to obtain information concerning their sociodemographic background and family history. Six candidates could not be reached with the provided contact data, and two additional candidates declined to participate in the study. In the data collection process, after each interview, the data were preliminarily analyzed to determine whether they contained new insights into the concept of stigma. After interviews with 16 relatives, theoretical saturation [67, 68] was reached (i.e., further interviews did not provide additional information); therefore, the sampling process was terminated at this point. One interview was conducted with a relative whose family member did not report mental health problems; this interview was not included in the analysis. Thus, this study is based on 15 participants, with whom 12 interviews were conducted.

### Participants

The sample comprised 15 relatives of former soldiers with a mental illness from five different federal states of Germany. We interviewed eight partners or spouses, five parents, and two adult children of former soldiers with mental health problems. In four of the 12 interviews, we spoke to more than one family member (mother and father, wife and daughter). The demographic characteristics of the interviewees are shown in Table 1.

**Table 1:** Demographic characteristics of the interviewees

Item	Gender		Age span
	Male	Female	
Spouses and partners	2	6	29–74
Parents	2	3	54–74
Adult children		2	18

All relatives reported that the former soldier suffered from service-induced mental health problems. Nine former soldiers were officially diagnosed by the German Armed Forces and had a recognized service-incurred disability status (Wehrdienstbeschädigung) because of a PTSD diagnosis. The other three former soldiers showed clear symptoms of PTSD (e.g., flashbacks, avoidance, intrusions, or hypervigilance) and had received treatment for them at some point in time; however, they were currently in the process of considering seeking help.

The former soldiers were between 26 and 64 years old (average age: 39 years). Nine of the soldiers were able to work and had a job. Three soldiers were unable to work.

The research team discussed the advantages and disadvantages of the inclusion of the three participants whose diagnosis was out of date, as they were currently not in treatment. Although it could not be verified whether these three former soldiers fulfilled the full diagnostic criteria at the time of the interviews, the researchers ultimately included them, as the relatives had reported several current relevant diagnostic criteria of mental illnesses in their family member. The decision to include these families also seemed to be justified by the fact that the researchers were also interested in stigma-relevant barriers to healthcare, and stigma against the label *mental illness* must be considered in this process.

### Data collection

In the data collection process, four researchers (KS, SD, LB, and MH) conducted detailed face-to-face narrative interviews [64, 65] with the selected family members. All interviewers had been trained in interview techniques and had participated in internal and external qualification and supervision measures. To conduct the interviews, two researchers visited the interviewees at their home or another location where they felt comfortable. One researcher conducted the interview, while the other researcher was responsible for the equipment, minute-taking and field observations. Before the interview started, the interviewees were provided with all relevant information concerning the aim, the scope and the methodology of the research. The researchers explained how the data would be evaluated and how the confidentiality of the data would be guaranteed. The interviewees provided written informed consent. In the interviews, the researchers did not specifically ask about stigma-relevant experiences; however, they used a narrative technique that always started with the following introduction: *“We are interested in your story of the time when your relative served in the German Armed Forces. We would like to know how it was for you when he/she served abroad and then returned home. How have you experienced this process?”* The interviewers let the participants talk about whatever was significant to them. If inconsistencies arose or the interviewees provided emotional responses, the interviewers encouraged them to elaborate more deeply on the emerging issues. The interviews were completed when the participants had nothing more to describe. After the interviews, the participants were given the opportunity to ask questions and were provided with the researchers’ contact information in case they subsequently had further questions.

All interviews were recorded as MP3 files and subsequently transcribed by an external transcription service that adhered to strict confidentiality regulations. Transcription was carried out using the guidelines of Dresing and Pehl [70], which specify that the oral version is to be transcribed verbatim, but dialects and function words are to be omitted, and punctuation was adapted to facilitate legibility. The complete body of the recorded interviews relevant for this study consists of 12 h and 55 min of recorded material, with the average interview lasting 65 min.

### Data analysis

The transcribed and anonymized data were analyzed using the thematic analysis approach [71], in which the data were iteratively coded using the program MAXQDA 12 (VERBI-Software-Consult-Sozialforschung GmbH, Berlin Germany). The objective of this coding process was to identify underlying patterns in the data that show how the families perceived stigma and what consequences it had for them. Thematic analysis may be subdivided into a four-step process: familiarization with the transcripts, identification of codes and themes, review of themes to identify structures and model construction [71, 72]. Following these steps, the researchers became immersed in the data through reading, re-reading and memo-writing [71] prior to conducting the actual coding. In the first coding step, three members of the research team (KS, SD, and LB) coded all interviews following an open-minded approach. At this stage, the researchers were not looking for specific links between the stigma-relevant codes or their theoretical classification; they were mainly interested in a complete coverage of all stigma-relevant remarks in the interviews. In this step, stigma-associated utterances were linked to codes that captured the meaning of these narratives. These codes summarized all stigma-relevant parts of the interviews and were used to index the data. The following example illustrates this coding step: Statement: “*…they told me that they did not want to have anything do with a mentally ill person because they could harm their kids.”* Code: *because they could harm their kids: mental illness = dangerous*. Inclusion and exclusion criteria for the codes were developed and defined, e.g., *mental illness = dangerous: contact is perceived as harmful*.

In the second step, the researchers analyzed the relations between the extracted codes and used these relations to form categories or themes [71] that represented these relationships. For example, *mental illness is dangerous* (code 1), *mental illness as malingering* (code 2)*, mental illness as freeloading* (code 3), and *mental illness as being weak* (code 4) were summarized in the theme/category *attitudes in mental illness stigma.*

In the third step, the research team analyzed and systematized these newly established categories or themes. In this stage, we also analyzed how the stigma-relevant categories were linked with existing theoretical frameworks and other relevant concepts, e.g., reactions of relatives and perceived consequences of the stigma experience. In this process, we drew conclusions that explained the relationships between the constructs in the dataset. The following example explains this step: Quote: “…And from one day to the next, when they found out that Michael* suffered from PTSD, they withdrew themselves from us because they do not want to be associated with mentally ill people…” (* name changed by the authors) was coded as follows:

- “*They withdrew themselves from us”* = code: social exclusion.
- *“Because they do not want to be associated with mentally ill people…” =* theme: reasons for withdrawal, subcode: mental illness.
- Conclusion: Mental illness is associated with social exclusion.

These three coding steps were iteratively completed several times and were constantly checked against the existing data. Triangulation and validation [73, 74] (i.e., considering different opinions and different theoretical frameworks, discussing different perspectives and questioning the researchers’ preconceptions) proved to be an important step toward following a reflexive and critical approach, as it also meant analyzing and considering the researchers’ pre-existing concepts and experiences to establish a critical distance to the data. All codes and themes, as well as the emerging connections between them, were re-analyzed in detail using a theory and investigator triangulation approach [73, 74]. The research team thoroughly discussed all inconsistent codes. When inconsistencies between the researchers’ opinions occurred, their respective perspectives on the data were further explored. In all cases, these inconsistencies could be explained by the researchers’ socialization in different professional fields (medicine and psychology). Therefore, some codes and themes were coded twice (one time with a psychological perspective and a second time with a more medical point of view).

In the final step, after coding, theory and investigator triangulation [73, 74] and systematization, a model was developed, which illustrated the relation between the stigma-relevant categories and themes, the families’ reactions to them and their effects on social relationships. The model development followed an iterative process whereby each draft of the model was again counterchecked against the data and changed if the data were not fully represented by the model. The final draft of the model was again communicatively validated [73, 74] by the research team.

## Results

The aim of this study was to determine how relatives of former soldiers suffering from service-induced mental illness perceive stigmatization. Furthermore, we aimed to analyze how these relatives reacted to this stigma. Third, we aimed to analyze which consequences of stigmatization they perceived in their social life and how it affected their healthcare utilization.

### Stigma experience of the relatives

Although the interviewers did not specifically ask about stigmatization, stigma was cited in all interviews. Overall, 101 different stigma codes were extracted. These codes were subdivided into two stigma types, including SBA and vicarious stigma, and relevant stigma topics, including mental illness stigma (47 codes) and former soldier stigma (54 codes).

#### Mental illness stigma

*Mental illness stigma* was directed at the interviewed family member (SBA, 14 codes) or the former soldier, leading to suffering and/or compassion in the interviewed family member (*vicarious stigma*, 33 codes). Thematically, the interviewees were mainly confronted with the following stereotypical attitudes: they were blamed for the illness (17 codes), the illness was not taken seriously (malingering, 14 codes), they were accused of being a freeloader (14 codes), or their family was perceived as dangerous (dangerousness, 2 codes).

#### Former soldier stigma

*Former soldier stigma* was coded when the interviewees were directly confronted with negative attitudes toward their relatives’ military past (SBA) or when they observed the former soldiers’ stigmatization and were indirectly affected by it (vicarious stigma). Former soldier stigma often interacted with one specific mental illness stigma attitude and its consequences: *blame*. The interviewees reported that they were confronted with the opinion that because the former soldier had joined the GAF voluntarily, the family could and should have known about the risks involved in the operations carried out by combat troops. As they had ignored this risk, they now had to take full responsibility for the illness and should not complain about it.

However, being a former soldier or being associated with one was also in itself stigmatizing, apart from the mental illness. The former soldiers had to face accusations of being murderers or mercenaries. Although this stigma was mostly directed toward the former soldier, the relatives were closely tied to the stigmatized individual and were therefore included in the stigmatized group (they belonged to a military family). In rare cases, female partners or spouses were directly accused of being a military wife, which was stigmatizing in and of itself. Table 2 illustrates how both stigmas, mental illness stigma and former soldier stigma, and their interaction were perceived.

**Table 2:** Perceived stigma ordered by stigma type and stigma topic

Stigma form		Sample quote	
		Stigma by association	Vicarious stigma
*Mental illness stigma* (47 codes)	Blame (17 codes)	*“She’s just a psychologist. Psychologist, but not specialized in PTSD, you know. And she draws up an expert opinion in which she says that it was, …, that WE are actually to blame for the fact that he has PTSD.”*	*"... it's your own fault. Why did you go there?"*
	Malingering (14 codes)		*“… well, as I said, with my parents, my family, it is complicated. Because he has two arms, two legs, he can take care of himself. He can work…”*
	Freeloading (14 codes)	*“… I think, there are always some colleagues who are rolling their eyes. Of course, when I’m sometimes ill…, they say: ‘She is only ill because of her husband’s illness.’”*	*“… Well, as his wife, I am also sorry if I know that he is treated differently just because he has this stamp on his forehead, ‘I am a PTSD soldier’. … And it always hurts him that he is not regarded as normal, but always as a PTSD soldier and that eyes are often rolled because he has special conditions. He is only allowed to work six hours… And I noticed it myself, when I picked him up there or brought him there… this eye rolling”*
	Dangerous (2 codes)	*“... and they avoided me, the mothers. Not so much the fathers, more the mothers. They had a problem with it, and they told me that they did not want to have anything do with a mentally ill person, because they could harm their kids.”*	*“… they don’t know how to deal with him, how to talk to him/ what they may or may not say… Somehow they’re all scared of him. I don’t know why. … he’s not a monster.”*
*Soldier stigma* (54 codes)		*“… But there are also people … who certainly don’t want to hear that you live with a soldier. It’s hard sometimes when it comes to the insults, which you must listen to: ‘Soldier’s whore’ and such things, which you get thrown at you. And that’s rough. It’s really rough, the hardcore pacifists. It’s not pretty.”*	*“…This is the dilemma nowadays, that society doesn’t appreciate soldiers any longer, and now, with professional armed forces, even less. They are murderers.”*
Interaction between *Mental illness stigma* and *stigma soldier*		*“…What I hear more often… that people say: ‘Why, he knows what he’s getting involved with, and they only go because they get a lot of dough anyway.’ … And then it’s their own fault, when they are sick, that’s their own fault.”*	

An analysis of the different family roles indicated that partners/spouses reported stigma most often, followed by parents. The interviewed children were not confronted with mental illness stigma; however, they expressed stigmatization because of their parent’s military past. Cohabitation was associated with more stigma. The two subtypes of mental illness stigma, vicarious stigma and SBA, showed a similar pattern of stereotyped attitudes. The only difference between the two types was that the stereotype *malingering* was expressed only in the form of vicarious stigma, i.e., the interviewees experienced how their relative was accused of malingering and suffered because of these accusations. We did not identify a gender difference in perceived stigma, as male and female interviewees expressed similar patterns of perceived stigma. The relatives’ stigmatization led to specific reactions (36 codes), which affected the social relationships of the family and healthcare utilization.

### Relatives’ reactions to stigma (36 codes)

When the relatives anticipated or experienced stigma, they showed four main patterns of reactions: nondisclosure, anger, acceptance or resignation and self-blame.

Nondisclosure (13 codes), one of the most prevalent reaction patterns, was cited by nearly all interviewees. It often occurred in connection with a potential stigma threat. The interviewees feared stigmatization and decided to keep the mental illness a secret: “… Well, we did not peddle this information* because we also noticed that… some had said: ‘Those who go out there, it’s their own fault if something happens” (*refers to the former soldier’s mental illness; explanation by authors). However, some interviewees also reported that when they opened up and told individuals about the mental illness, it helped them considerably. They felt understood, and many of the negative reactions they feared were not experienced.

Anger (13 codes) was also prevalent, although it presented in two different forms. Some interviewees expressed their anger and confronted the source of the anger, e.g., “*Those are the typical arguments that come, right? Yes. And then I can get on my knickers. Well, I won’t shut up, I must say. Then I already talk my frustration off my soul, so he never again makes any remark …*.” Other interviewees were angry but kept their feelings inside. The interviewees cited two reasons why they did not make a stand against stigmatization. Some interviewees indicated that they did not have the time- and/or strength-related resources to actively do something about this stigmatization (e.g., “… when you see that your husband comes home, suffers or takes out what he doesn’t dare to say there*, then takes it out on you, on my son, … and you then come to your limits… because I have so many hurdles to overcome …” (*refers to mental illness stigma at the husband’s workplace). Other interviewees believed that their actions could have negative consequences for the family (e.g., “*Sure, sometimes there are sentences like this: ‘You’ve been on voluntary duty, so you’ll have to deal with it.’ And… inwardly I could sometimes freak out and… would like to say something. But you don’t dare to do that as a wife because you think, then he has even more the stamp on his forehead, … ‘he is also under his wife’s thumb’, so you hold back relatively quickly”*).

Acceptance/Resignation (four codes) mostly referred to anticipated or experienced public stigma. The interviewees perceived this stigma as a given that could not be changed, e.g., *“… They go there because they get money. Period. And then it is their own fault if they, if they fall ill. Sure. They do it only because of the money. These stories are known, such stories. You hear them everywhere. That’s just the way it is.”*.

Self-blame (three codes) was cited only by mothers of former soldiers. They blamed themselves for not preventing their adult child from joining the military and going on a mission abroad, e.g., *“... he joined the GAF when he was very young because he had no perspective in civilian society. That’s why we sent him to the GAF. I don’t know if this was a good idea. Sometimes, I blame myself for it a bit.”*.

There was no difference in the relatives’ reactions between the two subtypes vicarious stigma and SBA*;* all four reactions were shown for both subtypes. The analysis of the different family roles showed that *nondisclosure* and *anger* were omnipresent across all family roles. *Acceptance and resignation* were mainly found in partners/spouses and parents, whereas *self-blame* was reported only by mothers.

### Consequences of stigma (77 codes)

The analysis of the perceived consequences of stigma showed effects in two different spheres of life: social relationships (65 codes) and healthcare utilization (12 codes). Most consequences were reported in relatives’ **social relationships.** Relatives reported four major consequences in this area: loyalty conflicts, conflicts at work, social exclusion and self-segregation.

Loyalty conflicts were mainly experienced by partners and spouses who were confronted with the expectations of other family members or friends that conflicted with their spouse’s/ partner’s wish to be understood and helped, e.g., “… And recently she* has demanded of him to be normal. ‘How long should this last?’ These are the remarks that come from my family and from my father’s side, who says to him, ‘Pull yourself together. It has to get better.’ Or he asks me how long I can accept this, how long I want to stay with him” (*interviewee’s mother; explanation by authors). These conflicts required energy, which was a sparse resource, and led to less social support for the family because the family did not want to confront these expectations; thus, they avoided these topics and, in some cases, avoided contact altogether.

The second consequence in social interactions was conflicts at work. As the former soldiers required care or could not fulfill responsibilities in the family, the interviewees, mainly the spouses and partners but sometimes also the parents and children, had to step in. This meant that they had to reduce their hours at work or take time off. The workplaces reacted differently to these changes in commitment. Some workplaces were understanding; however, in other cases, conflicts arose, e.g., “… Yeah, and then a colleague said to me, ‘Well, when you do not want to work on Fridays*… and I say… whoever knows me knows that I would not even consider this.” (*This situation occurred after the interviewee took a Friday off from work to visit her son in the hospital; explanation by authors). For the interviewees, these conflicts were associated with additional stress, as they described their work as a place where they could tune out problems at home. Colleagues were often the ones to whom the interviewees confided their problems. This meant that these conflicts in their work environment robbed them of a quiet place that had not been impacted by their relative’s mental illness and had been an important source of social support.

The third consequence for the families’ social network was social exclusion, i.e., the termination of contact due to the mental illness of a family member. For example, “… We lost, well, we also lost friends. We lost many friends. One friendship, they were our close and best friends… And from one day to the next, when they found out that Michael* suffered from PTSD, they withdrew themselves from us because they do not want to be associated with mentally ill people” (*interviewee’s husband; name changed by the authors). However, mental illness was not the only reason for social exclusion. In rare cases, the interviewees were also excluded simply because of their link to the GAF, e.g., *“… they do not like the GAF. And then… they did not want to have anything to do with us.”*.

Social exclusion also occurred in the form of self-segregation, i.e., the families avoided social contact because they did not want to be confronted with stigma, or they perceived that social contact had become complicated because of a stigma threat. For example, “… Sometimes, I don’t even know what to answer, right? … Then you’re just stammering around, right? … You always must worry somehow, and you always must remember, to whom did you tell what? Who knows it*? Who doesn’t know? It’s kind of a crazy thing, isn’t it?” (*refers to the husband’s service-induced mental illness; explanation by authors).

All four consequences, including social exclusion, conflicts at work, loyalty conflicts with family and friends and self-segregation, reduced the social resources on which the families could potentially count. It also meant that relationships were perceived as strained and difficult because of experienced or anticipated stigma. Overall, the interviewed relatives perceived these consequences as a decline in the quality and size of their social relationships.

**Fig. 1:**
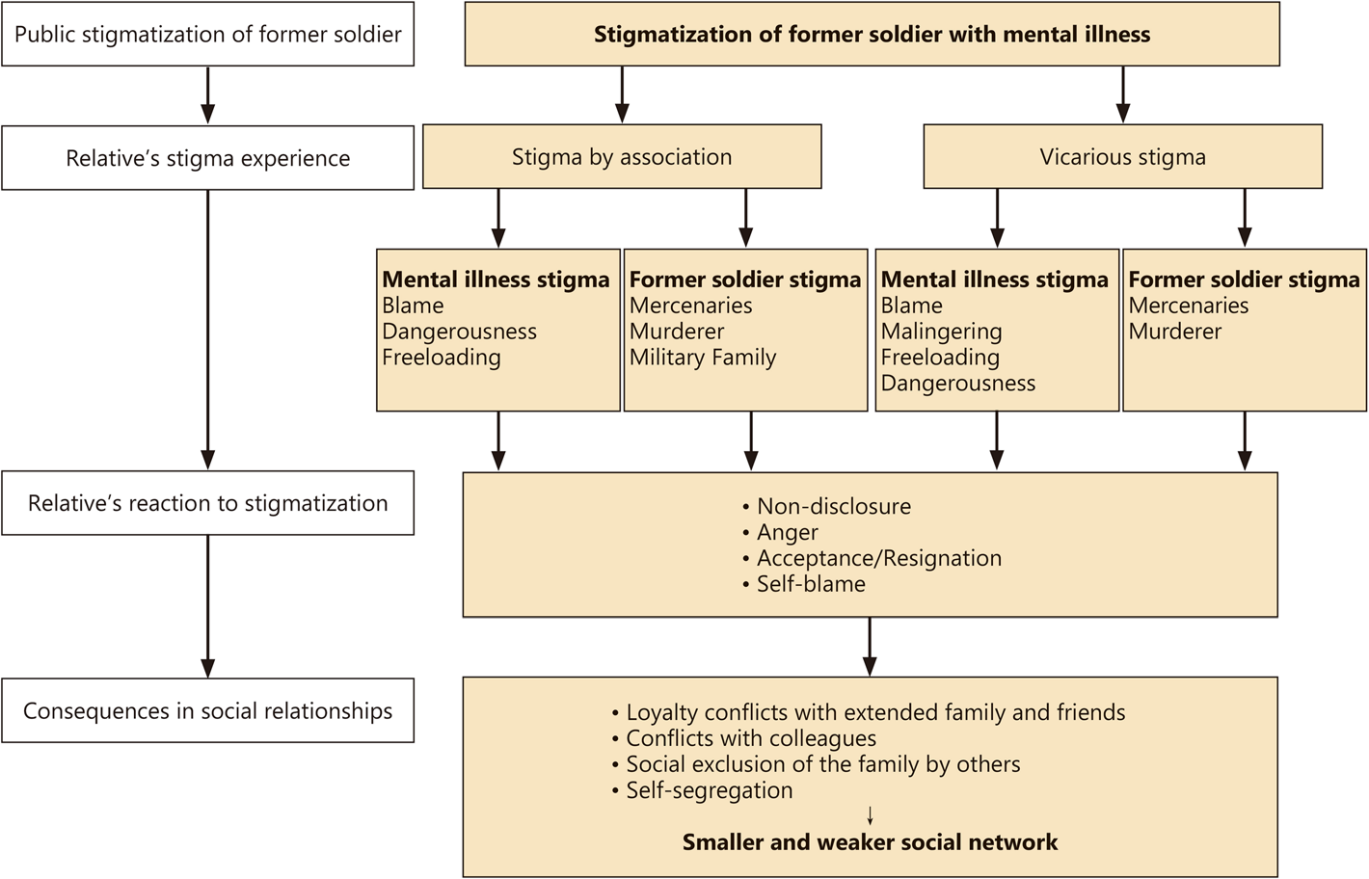
Impact of stigma on social relationships perceived by interviewees

The following illustration shows the relationship between the different types (SBA and vicarious stigma) and forms (mental illness and former soldier stigma) of stigma, the relatives’ reactions to it and its consequences for the families’ social network (Fig. 1).

A second sphere of life in which the interviewees experienced an influence of SBA and/or vicarious stigma was the **healthcare utilization** of the former soldier or relatives (12 codes). Our analysis showed three different pathways by which stigma interacted with healthcare utilization:

1. Vicarious mental illness stigma directly influenced former soldiers’ healthcare utilization when they were accused of malingering by healthcare personnel, e.g., “…he also often has the feeling that he is not taken seriously there* at all… After so many years, he was called a malingerer there… But it was such a setback for him. It was such a stab in his soul. He couldn’t take that… These are the little things that make life difficult for ME…” (*referring to a military hospital; explanation by authors). The relatives perceived the former soldiers’ stigmatization in healthcare settings as highly problematic, as it often required considerable effort on their part to convince the former soldiers to seek help in the first place. Some interviewees reported that the former soldiers did not perceive themselves as needing healthcare, even though they showed clear symptoms of mental diseases. The former soldiers’ internalized value system of being self-reliant and strong made their decision to seek help very difficult. The relatives often had to actively ask them to go and see a doctor, e.g., “…*I’ll go along with anything, but not if you don’t seriously try to change it… Please go to therapy.”*.
2. Another form of stigma that interacted with healthcare utilization was vicarious former soldier stigma. As the former soldier’s healthcare was often initiated by the relatives, they were also the ones who were confronted with this form of stigma in a civilian setting. When looking for therapy, they found that the military background of their relative’s mental illness was stigmatizing, and it was therefore difficult to find healthcare personnel who did not endorse this stigma, e.g., “…*… And then find someone to help him, not that he comes to someone who says,” “Yes, it’s your fault” or “You got a lot of money” or “You’re the killer.”*.
3. The third pathway identified was an indirect pathway. The interviewees reported that the overall demands with which they had to cope were simply too high and robbed them of the time and energy to take care of their own health problems. SBA and vicarious stigma contributed to this burden and were therefore indirectly associated with their lack of healthcare utilization, e.g., “*… I have to go to work. I still have to look after my son, actually after my husband as well… and this year… I have unfortunately stopped my therapy… I have discussed this with my therapist, that I currently have no energy to talk about my own problems… because too many appointments are buzzing around in my head… It’s just difficult… As a relative… you want to know that your husband is well… And if you notice that he is also not well… because he is bullied… that made my life difficult.”*.

## Discussion

### Stigma by association and vicarious stigma

This study was the first investigation to analyze SBA and vicarious stigma in relatives of former GAF soldiers with a service-induced mental illness. It replicated many findings of similar research. The relatives in this sample perceived mental illness stigma of being *blamed*, being accused of malingering and freeloading and being dangerous, which is well-documented in international studies in military contexts [51, 52, 75, 76]. The first studies in the context of the GAF showed similar attitudes toward active [60] and former soldiers [61].

In addition to replicating previous research findings, this study produced new results: apart from mental illness stigma, the relatives also experienced a different form of stigma, former soldier stigma, which impacted several spheres of their lives. This stigma has previously been found among former soldiers themselves, whereby it interfered with their healthcare utilization [61]. This study shows that it seems to play a role in their families. This stigma, which has not been reported in any other military context apart from the GAF, might be associated with the GAF specifically. As the public opinion on the military operations of *German* soldiers abroad might still be shaped by German history, the soldiers and their families face negative reactions from parts of the German population when they disclose the military service of their family member. This result is supported by research into how the German population perceives the GAF and its participation in military operations abroad. A recent study by the Centre of Military History showed that only 50% of the German population believed that GAF members have a positive reputation in Germany [77]. A significant segment of the participants (26%) did not consider the GAF as an integral part of German society, and 38% were not grateful for its service. Fewer than half of the participants supported the involvement of the GAF in international deployments (from 41% supporting the International Security Assistance Force (ISAF) in Afghanistan to 32% supporting the United Nations Interim Force in Lebanon (UNIFIL)). In a similar survey, 46% of participants expressed doubt, 32% fear, 22% anger and 12% contempt toward the GAF [78]. These findings show that German (former) soldiers and their families cannot always expect appreciation and respect from large portions of civilian society. In contrast, they must fear being stigmatized for their service.

The second important finding of this study is that the two stigma topics of mental illness stigma and former soldier stigma were interrelated. In particular, the mental illness stigma of *blame* was closely linked to the military past of the veterans. In previous research, blame mostly referred to the onset of the illness directly (the relatives were blamed for it) [18, 33], whereas in our study, the onset of the mental illness was attributed to the military operation abroad. The relatives were blamed or blamed themselves for not having intervened, for not having prevented the former soldiers’ military participation, or even for profiting from it financially. This aspect could be explained by attributional processes. According to Weiner [79], different attributions for the causes and controllability of an individual’s illness lead to different perceptions about the individual’s responsibility for the illness. If an individual is perceived to be in control of the causes, responsibility for it is inferred. Research [80] shows that individuals who are perceived as being responsible for their illness elicit more negative emotional responses, such as anger or accusation. Following Weiner [81], responsibility requires human agency, i.e., the individual affected by a negative result (here, a mental illness) must contribute to the outbreak by doing something. If this contribution can be controlled by the individual affected, negative responses by other individuals can be expected [81]. In our study, the former soldiers, who had joined the GAF voluntarily and gone on missions abroad, were fully aware of the implied danger. Their families had not intervened and had potentially profited from the financial compensation for their missions. Their voluntary decision to join the GAF and go on a mission abroad could therefore be viewed as an internal controllable action by the former soldiers and their families; they were thus perceived as responsible for the results. This might explain why the former soldiers and their families were blamed for the mental illness.

However, we are not aware of stigmatization of firefighters or rescue workers, who also knowingly expose themselves to the risk of traumatic events. We assume that the image of the job or institution might play a role in this association. Firefighters are needed, and their job enjoys a very high reputation in the German population [82]. This reputation might function as a mitigating factor [81] that reduces or even lifts the responsibility for negative outcomes experienced by firefighters on the job. Unfortunately, this cannot be said about former soldiers of the GAF, whose image in German society is ambivalent and partly outright negative [77]. This partly negative image of the GAF might contribute to the attributional processes involved and lead to negative reactions. Given that the former soldiers’ mental illness was service-induced and military service is associated with a negative reputation, the former soldiers’ mental illness is perceived as a result of an internal, controllable action, thus leading to blame and negative reactions. Families who had supported this mission or had not prevented the former soldier from participating in it might be perceived as having indirectly contributed to this illness.

The analysis of different family roles, gender and cohabitation did not show surprising results. The stigma experience varied across different familial roles, with spouses and partners reporting stigma most often and adult children reporting it the least. This result might be confounded with cohabitation: The partners and spouses lived with the former soldier, whereas this was not the case for all adult children. There were no gender-related differences. These findings are in line with previous research [63]; however, the small sample size (e.g., only two children, only two male partners) must be considered in the interpretation of these findings.

### Reactions to stigma

The families were aware of stigmatizing attitudes pertaining to both mental illness stigma and former soldier stigma, and they reported that these attitudes shaped their behavior. Three of the four reactions identified in this study (nondisclosure, anger, and acceptance/resignation) are well known from previous research [8, 10, 18, 33] and were similar for mental illness stigma and former soldier stigma. The very different reactions to similar stigmatizing attitudes could be explained by the findings of Corrigan and Watson [83], who stated that social stigma might be associated with different reactions to it. Van der Sanden et al. differentiated between problem-focused and emotion-focus reactions [8, 10, 25] and found that relatives’ reactions to stigma can mediate its effects [10]. As in previous research [8, 10, 50], the relatives in our study showed both types of reactions: they used problem-focused approaches, e.g., confronting stigmatizers with rightful anger, and emotion-focused coping, e.g., nondisclosure, resignation and self-blame. The analysis of the relatives’ reactions showed that their reactions had a mediating function. The relatives who reacted with nondisclosure to stigma threat avoided the stigma in the short run. In the long run, however, they deprived themselves of helpful social resources, which made these strategies less successful [8, 10]. As shown in other studies [83, 84], the relatives who, for example, finally opened up and asked for help or confronted a stigmatizer by showing their rightful anger felt empowered and reported that the stigma was substantially lower than anticipated. Similarly, several interviewees stated that they had been afraid to participate in the interviews; however, once they talked about their issues and were not stigmatized, they felt substantially better. Therefore, low-threshold services that allow relatives to talk about their problems, for example, self-help groups, might show them that nondisclosure and resignation are not very helpful strategies [8].

The fourth reaction to stigma, self-blame, was reported by parents, particularly mothers. Research shows that parents are often blamed for their children’s disease, lack of adherence and slow recuperation [18]. Although self-blame can be viewed as the internalization of the mental illness stigma blame, we found differences in their underlying processes. In our sample, both blame and self-blame can be described by the following attribution process: The relatives were blamed by other individuals and/or blamed themselves, particularly for the onset of the mental illness, because they were attributed responsibility for the former soldier’s decision to join the GAF and go on a mission abroad. However, there were also differences in the two attributional processes. Blame was mainly associated with the negative image of the GAF (former soldier stigma; because of negative attitudes toward GAF participation in military operations abroad, deployment abroad did not function as a mitigating factor. Therefore, the relatives were perceived as responsible for the illness and were consequently blamed for it). In contrast, we did not identify this pattern for self-blame. The bad reputation of the GAF (former soldier stigma) was not associated with self-blame. However, self-blame was associated with the fact that the parents had supported their child’s decision to join the GAF and go on a mission abroad or they had not intervened to prevent it. Therefore, they blamed themselves for not having prevented the illness, which they perceived to be caused by the military operation. Whether this mission abroad was viewed as good or bad by the parents or the general public seemed to have no influence on the parents’ reaction of self-blame. From this perspective, similar results should be found in parents of firefighters, for example. In contrast to former soldiers, firefighters have a positive image in society, and their job is viewed as very important. This could be a mitigating factor [79] in the attribution of responsibility; however, their parents may still blame themselves for the onset of illness. However, we are not aware of research on this topic. Further research into these processes should address these questions.

### Consequences of SBA and vicarious stigma

Most consequences of SBA and vicarious stigma for the **social networks** of the relatives in our study, such as loyalty conflicts with family and friends, as well as conflicts at work, social exclusion and avoidance of contact, are in line with findings of previous research [8, 10, 18, 19, 21, 33] and are associated with mental illness stigma. However, former soldier stigma also played a major role in social exclusion and self-segregation: some families felt isolated because of their specific issues and therefore had problems integrating this part of their life into the civilian environment. They had civilian friends and colleagues; however, when it came to problems related to the family member’s service-induced mental illness, they attempted to stick to (former) military contacts. This tendency has been identified in previous research [41] that shows military families doubt whether civilians can understand the stressors associated with military service, deployment and reintegration; they therefore share their problems with other military families [41]. In our study, however, the former soldier stigma played an aggravating role: the relatives feared being confronted with negative attitudes toward the GAF’s military operations abroad, which were viewed as the source of the mental illness. Therefore, they spoke only with military families about their problems; they did not share their issues with civilian friends, colleagues or, in some cases, other family members. In some cases, this led to a compartmentalized way of life for these families. They had the civilian compartment, where they did not talk about the service-induced mental illness or its consequences for the family, and the (former) military compartment, where they could open up. This might also explain why help offered by the GAF in the form of seminars and weekend retreats for military families was highly appreciated, as these services offered a safe environment for them to talk and share their problems without fear of stigma. Unfortunately, the families reported that these seminars were a scarce resource and were mainly offered to couples. Similar seminars for adult children and parents were lacking. Furthermore, these seminars did not help them open up in the civilian environment. Marek et al. [41] suggest that civilian healthcare providers and social workers should consider these concerns and show “respectful curiosity and acknowledgement that you are an outsider” (pg. 448) to increase rapport with these families. Furthermore, anti-stigma campaigns should not only address mental illness stigma but should also consider the negative image of the GAF and its influence on military families.

A second sphere of life in which the relatives perceived the impact of SBA and vicarious stigma was **healthcare utilization**. Our study showed that former soldiers, as well as their relatives had to cope with intolerance, as well as a lack of understanding and knowledge in healthcare institutions. These findings are in line with results from previous research [8, 10, 84]. Our study also confirmed that relatives are more aware of relevant symptoms than former soldiers themselves and that they would be willing to seek help for both themselves and the former soldier, despite stigma [41]. However, former soldier stigma was perceived as an additional stigma threat in civilian healthcare settings for both the former soldier and the family members. It was associated with healthcare avoidance and more difficult access to care. In Germany, former soldiers have access to help in military hospitals. However, their relatives must use civilian healthcare facilities. Thus, as there is limited capacity in the few military hospitals and local healthcare is easier to reach for families, it should also be possible for these families to find efficient help in local civilian healthcare facilities. However, current facilities are often not aware of these families’ needs. The relatives in our study felt left out of the team in healthcare. Healthcare, particularly in civilian settings, should therefore be prepared for the special requirements of these families. Marek et al. [41] propose that the partners’ perspective might be an important input for the diagnosis and treatment of mentally ill service members and that their treatment might benefit from a more systemic care perspective, one that includes the whole family [41]. Decreasing stigma-related stress factors might therefore not only be important for the family life of veterans with a mental illness but also indirectly contribute to the veteran’s recovery [41].

### Strengths and limitations

We believe that this study has several strengths. First, it contributes to the limited body of research on the families of former GAF soldiers who suffer from mental health problems. Second, it is the first investigation to analyze how these families are affected by SBA and vicarious stigma, how they react to stigmatization and how their social relationships and healthcare utilization are affected by stigma. This study provides a detailed, descriptive overview of SBA and vicarious stigma. Their effects on the social networks of these families are shown and illustrated in a model, which indicates how relevant variables are interrelated with each other. Furthermore, the study provides initial insights into how SBA and vicarious stigma interfere with the healthcare usage of these military families. Should further research replicate our results, these findings might support the development and implementation of measures to fight stigmatization.

Although the study used a qualitative design and therefore had only a small sample, it is this qualitative design that enabled us to obtain deep insights into the experiences of different types of relatives: spouses, partners, parents and adult children. High quality standards and strict rigor in the scientific process were of major concern and were guaranteed by an ethical, theory-based and iterative approach in sampling, data collection and data processing. The researchers applied a multidisciplinary perspective, as the research team consisted of medical doctors and psychologists with and without clinical experience, with and without a military background, and with and without deployment experience. This team enabled different perspectives on the data. In addition to team-based triangulation [73, 74], peer consulting, consensus building and presentations of preliminary results to internal and external experts, all team members regularly participated in methodology training, research workshops and external research supervision.

Nevertheless, several weaknesses should be considered. This study was conducted using a qualitative, cross-sectional design, which did not allow for generalizations or conclusions on causation. Furthermore, we interviewed relatives who contacted us via electronic media. Therefore, we could not reach relatives who do not use these media. We must also consider response bias [85], which indicates that a special group of relatives, namely, those who had the time- and strength-related resources for such an interview, participated in our study. It is possible that other individuals simply did not have the strength to make this effort. Moreover, anticipated or experienced stigmatization could have prevented relatives from participating in our study. This indicates that the effects of SBA and vicarious stigma may be underestimated in this study. Furthermore, it may be argued that relatives without stigma experiences might be less motivated to contribute to research on it.

It should also be considered that memory bias might play a role, as the interviewees’ reports covered larger time spans, and their experiences at a certain point in time were influenced by later events. Furthermore, it should be considered that we did not use standardized diagnostic methods to include or exclude participants, which decreased the comparability of our results with those of other studies. As all former soldiers had treatment experience because of PTSD, it may be assumed that they had been diagnosed at some point in time.

Although we found some variance in how specific family members perceived stigma, the limited number of interviews might diminish the generalizability of the findings. This same restriction applies to gender differences.

## Conclusions

Our results stress that stigma affects not only active and former soldiers but also their families via SBA and vicarious stigma. Social relationships with extended family and friends, as well as colleagues play a major role as a social resource for these families. The interviewed families perceived that their social resources were negatively influenced by SBA and vicarious stigma: their social networks decreased, and the quality of their relationships declined. The relatives also perceived a negative influence of stigma on their and the former soldier’s help-seeking behavior.

Therefore, family-centered anti-stigma campaigns are urgently needed. The needs of relatives should be included in measures to decrease stigma. These measures should not only disseminate realistic information regarding mental illnesses but also create opportunities for contact between mentally ill individuals and the general population, which has proven to be an efficient intervention strategy [86–88].

A significant finding of this study was that the families were stigmatized not only because of the former soldier’s mental illness but also the former soldier’s military background (former soldier stigma). Therefore, in addition to fighting mental illness stigma, anti-stigma campaigns must work to improve the reputation of the GAF in the general public. Initial studies regarding the economic profitability of anti-stigma campaigns suggest that these campaigns are also a good financial investment [89].

Because this study was the first investigation to research SBA and vicarious stigma in GAF families and it was exploratory in nature, further research should address both forms of stigma, namely, mental illness stigma and former soldier stigma, as well as the potential interaction between them. Our study shows that the interviewed families needed help and that they knew exactly what type of help they needed. It is time to listen to them.

## Abbreviations

GAF: German Armed Forces
ISAF: International Security Assistance Force
PTSD: Post-traumatic stress disorder
SBA: Stigma by association
UNIFIL: United Nations Interim Force in Lebanon
